# Pathology and Practice of Medicine

**Published:** 1861-11

**Authors:** Richard J. Dunglison

**Affiliations:** One of the Physicians to the Pennsylvania Institution for the Instruction of the Blind; Honorary Local Secretary to the Sydenham Society of London, etc.


### PATHOLOGY AND PRACTICE OF MEDICINE.

BY RICHARD J. DUNGLISON, M.D.,

ONE OF THE PHYSICIANS TO THE PENNSYLVANIA INSTITUTION FOR THE INSTRUCTION OF THE BLIND; HONORARY LOCAL SECRETARY TO THE SYDENHAM SOCIETY OF LONDON, ETC.

I.—*Statistics of Prisoners: their Mental Condition and Diseases.* By J. B. THOMSON, L.R.C.S., Edin., Resident Surgeon General Prison for Scotland at Perth. (Edinburgh Medical Journal, August, 1861, p. 136.)

In this interesting continuation of previous papers on the mental condition, etc., of prisoners, inquiry is made bearing upon two questions of great interest to medical psychologists, viz.: To what extent are crime and mental disorder allied, and what are the class-influences at work in the factorship of insanity.

The author thinks it has been proved in the preceding papers—1. That criminals are liable to numerous physical disorders and diseases, especially of the nervous system. 2. That as a class, criminals, compared to the common population, are generally of an inferior average intellect. 3. That (without including criminal lunatics) fully more than 12 per cent. of the average daily population are noted and reported by the medical officers of the General Prison of Scotland as laboring under imbecility, suicidal tendencies, epilepsy, or milder forms and degrees of mental weakness.

Dr. Thomson confines his remarks in this article to *Criminal Lunatics*, as illustrative of the mental condition of criminals as a class, and their liability to diseases and disorders of the mind. The subject may be presented under the following aspects:—

I. Of prisoners in the General Prison of Scotland who *became* insane while undergoing their sentences of imprisonment, and the proportion of these to the average daily population of the General Prison.

II. Of prisoners in the whole of the prisons throughout Scotland, and the proportion who *became* criminal lunatics out of the whole average daily prison population of Scotland.

III. Of the criminal lunatics actually *existing*, and their proportion to the number of the criminal population compared with the proportion of lunatics to the general population.

Without entering into minutiæ, we may refer to the conclusions drawn by the author.

Comparing the criminal with the common population of Scotland, and the proportion of lunatics to each, we have the following striking equation:—

| | | | |
|---|---|---|---|
| Entire population of Scotland as in 1851, | Insane in 1860, / Sane, | $\frac{8,084}{2,888,742}$ | $= \frac{1}{356}$ |
| Entire prison population of Scotland, annually averaged, | Insane, / Sane, | $\frac{85}{2,281}$ | $= \frac{1}{65}$ |

Dr. Thomson states that, with the exception of paupers, he knows of no statistics of any class of the community by which to make a comparison with the criminal class; but it is obvious that insanity leads to pauperism, and *vice versâ*, so that the proportion of lunatic paupers to the pauper class, or to the general population, could not afford any satisfactory basis of comparison.

The foregoing statistics bring out the following results:—

| | |
|---|---|
| In the *General Prison of Scotland* the proportion of prisoners who became insane during the quinquennial period, 1856—1860, was . . . . . . . . . . . . | 1 in 118 |
| During the decennial period, 1851–1860 . . . . . . | 1 in 175 |
| In all the population of the Scottish prisons . . . . . | 1 in 228 |
| Proportion of *existing* criminal lunatics compared to the whole prison population . . . . . . . . . . | 1 in 65 |
| Proportion of lunatics in the general population of Scotland . | 1 in 356 |

The conclusion from this analysis is, that criminals, as a class, are extremely liable to mental diseases, and show a high ratio of insanity compared to the civil population.

These facts are drawn from what has been actually ascertained regarding the amount of criminal lunacy; but there is no doubt that a large amount is unknown, in consequence of ignorance of the number of liberated prisoners who lapse into insanity, and whose history, after passing into asylums, is not traced to be of criminal origin.

So far as the 139 cases given go in regard to *age*, it would seem that the liability to criminal insanity was at its maximum from the age of 20 to 30; that it rose in the earlier periods of life, and declined with advancing years. Esquirol's experience led him to consider that insanity increased progressively after maturity. Dr. Thurnham's cases, amounting to 5122, showed the greatest liability from 20 to 30; the Hanwell statistics of 1766 cases indicated the critical period to be betwixt 30 and 40—Dr. Thurnham's statistics corresponding with those of our criminal lunatics.

In these 139 we note the following number of the two sexes:—

| | |
|---|---|
| Males | 95 |
| Females | 44 |

*The males are more than twice the number of the females.* This is very remarkable, and different from the liability of the sexes as declared by statisticians in regard to the general population. Of Bethlehem Hospital we have a table of the annual admissions of lunatics extending over thirty-eight years, which gives as under:—

| | |
|---|---|
| Males | 3511 |
| Females | 5407 |

Indicating upon a large scale the greater number of lunatic females. In 1858, the Lunacy Commissioners for England stated the proportion of insane of the two sexes to be:—

| | |
|---|---|
| Males | 8247 |
| Females | 9165 |

And in the Third Report of the General Board of Commissioners in Lunacy for Scotland, we learn that the results of 1858 and 1859 tend to show that females are more liable to lunacy than males.

The author offers no explanation of this singular exception from other statistics of lunacy, that the males preponderate in numbers so much over the female criminal lunatics.

II.—*Mode of Fatal Termination of Fifty-Two Cases of Typhoid Fever.* Communicated by Dr. Bristowe to the Pathological Society of London. (Edin. Med. Journal, August, 1861, p. 156.)

In the 52 cases cited by Dr. Bristowe, the modes of death were as follows: In 8 instances the patients died before ulceration had been thoroughly established in the diseased Peyerian patches, and evidently from the uncomplicated effects of the typhoid poison. In 9 cases death occurred late in the disease, and when many of the ulcers had become more or less perfectly cicatrized, from perforation, from relapse, or from pneumonia, pleurisy, abscess, or debility dependent upon other causes. In the remaining 35 examples, the fatal termination took place at various periods between the above limits, either, as in the first class, from the direct influence of the disease itself, or from this associated, as in the second class, with diarrhœa, intestinal hemorrhage, bed-sores, pulmonary mischief, or perforation and peritonitis. In 15 of the total number of cases, death took place from peritonitis, the result of perforation of the intestines. In

9 cases perforation occurred during the process of separation of the sloughs; in 5 after the slough had become completely detached; and in 1 while cicatrization, excepting only the ulcer perforated, was progressing.

III.—*On Sexual Limitation in Hereditary Disease.* By WILLIAM SEDGWICK. (Brit. and For. Med.-Chir. Review, April, 1861, pp. 477–489, and July, 1861, pp. 198–214.)

In the words of Mr. Sedgwick, the hereditary transmission of disease is a subject for inquiry so vast and so little understood, that any fresh facts likely to throw light on only one of its subdivisions, cannot fail to be of some use, and to win perhaps for the whole subject a greater consideration than it has yet received. Hitherto but little notice has been taken of sexual limitation in hereditary disease, though it is of so constant and so regular an occurrence as not to be referable to mere coincidence. We may briefly cite a few of the illustrations which Mr. Sedgwick has gathered together, as elucidative of such transmission.

I. In *cutaneous affections*, allusion is made to examples of ichthyosis hereditary for three generations, the disease not being developed in the second generation, but reappearing in the third, the females alone transmitting the disease, but its appearance being restricted to the males; and of the disease being hereditary for four successive generations, and strictly limited to the male sex, two brothers having seven sisters who were free from it. The result of further researches, now being carried on, will probably add to our knowledge of the development and transmission of hereditary diseases of the skin.

Among the hereditary diseases of the skin, in which sexual limitation prevails, might be included the so-called *hemorrhagic diathesis*, which is said to be so common in the west of Germany, and in the Rhenish provinces, that in some families scarcely a single male arrives at maturity. The writer's main arguments for considering this peculiarity of the system, which is due, perhaps, to congenital malformation of the solids, rather than to any altered condition of the blood itself, as a hereditary affection of the skin, are: 1st. The hereditariness of the affection itself, extending to as many as five or more generations of the same family. 2d. The sexual limitation of the disease, the males being affected far more than the females, although the latter have a greater tendency to hemorrhage than the other sex, may be referred to as an instance of sexual preference in disease; but in some of the cases it is recorded, that while the males alone have suffered from the disease, the females alone have been able to transmit the disease, passing from the grandfather, through the daughter, to the grandson. 3d. The association, in some cases, of the hemorrhagic diathesis with hereditary rheumatism, a disease essentially characterized, if not initiated, by perverted action of the skin; an illustration being afforded by the prevalence of the united diseases for five generations, affecting exclusively the males, while the females, although they have never presented the same tendency to either disease, have, without exception, transmitted both to their male children. 4th. In some of the cases recorded, there has been a distinctly hemorrhagic eruption on the skin.

II. In *affections of the organs of the senses*, those of sight and hearing are alone discussed by the writer.

1. In *cases affecting sight*, we find instances of albinoes in a family, three males in one set of six children, the first and fifth being girls, without any such appearance, the second and sixth boys and albinoes; the third and fourth, twin boys, one an albino: of hereditary albinoism attacking a family in various generations, in one, especially, of which, there were four albino daughters out of a family of three sons and nine daughters.

In another instance, for three generations, the eyes have resembled in miniature the markings on the back of a tortoise-shell cat, restricted exclusively to the female sex. The same peculiarity occurred in every member of a family of fifteen brothers and five sisters, the inheritance being derived from the mother.

Instances of hereditary differences in the color of the two irides, and in the presence of coloboma iridis or cleft iris are also given; in the latter case the defect being transmitted from the mother's father without its being shared in by the mother, and her three daughters being free from it, though two of the three sons had it, the inheritance extending over four generations at least. Congenital absence of the iris, congenital opacity of the cornea, cataract, amaurosis, microphthalmia, congenital absence of the eyes, defect of muscular apparatus in the eye,—in one case of squinting the defect being limited to five boys in a family of ten, in which there were five girls—have also been hereditary and sexually restricted. In functional defects of the organ of vision, hemeralopia, nyctalopia, myopia, etc., have been limited in the same way; in one case of hemeralopia, the defect prevailing for two centuries, eighty-five members, belonging to six successive generations of the same family, having been hemeralopic, the defect being transmitted more by the women than by the men: in one instance of short-sightedness the affection was transmitted from the grandfather to the father, and from the father to the two sons.

Color-blindness is an illustration not so much of sexual limitation as of sexual preference. In one case mentioned, for instance, in five families in different generations, the total number being sixty-one, of whom thirty-two were males and twenty-nine females; of the former nine-sixteenths were affected, and of the latter only one-fifteenth. This defect of color-blindness seems to be almost exclusively limited to the male sex, for an analysis of two hundred cases and upwards shows that the proportion of males affected is nine-tenths of the whole. Yet where it has primarily affected the female sex, its sexual limitation is complete, as in an instance in which the defect occurred in thirteen individuals belonging to five generations of one family, all of whom were females; and several illustrations of a similar kind are mentioned by Mr. Sedgwick.

2. In hereditary diseases of the *organ of hearing*—which are, indeed, occasionally associated with defects of that of vision—striking cases are also recorded, the defect of color-blindness being sometimes associated, for example, with a corresponding inability to distinguish musical sounds; but the latter form of defect is not apparently peculiar to either sex. Deaf-dumbness sometimes appears to have a sexual limitation, as in the following illustrations: a carpenter at Paddington is the father of six children, three boys and three girls; the three boys have no imperfection of speech or hearing, the three girls are deaf and dumb. In a family of thirteen in the County of Sligo, five were males, none of whom presented any defect; eight were females, of whom seven were born deaf and dumb. The writer then proceeds to show by statistics, that deaf-dumbness is not hereditary in the common acceptation of the term, but only in that wider sense in which we apply it, in consequence of several members of the same family, their cousins, or other collateral branches, being similarly affected.

III. In *phthisis*, the same sexual limitation has been observed; of 669 male and 341 female patients, suffering from that disease, admitted into the "Hospital for Consumption and Diseases of the Chest," 18·2 per cent. of the former and 36·2 per cent. of the latter were hereditary, and while the father transmitted the disease to his sons in 59·4 per cent., and to his daughters in only 43·5 per cent., the mother transmitted it to her sons in 40·6 per cent., but to her daughters in 56·5 per cent.

In elucidation of similar points of sexual limitation in hereditary disease, cases are recorded of hereditary *imbecility*, limited to the female line; of hereditary *twins* from daughters in a family in which the wives of sons have had but single children; hereditary *malformation of the hands*, limited to one sex, etc. Sexual limitation in the transmission of disease is considered by Mr. Sedgwick to be more common in females than males. In conclusion, we may state that what the author of this paper seems to contend for is, that when the limitation referred to does occur, the tendency of the restriction to become hereditarily attached to one sex is sufficiently decided to merit special notice, and that, although the disease may show itself in the opposite sex in succeeding generations, yet there is always a marked preference for that in which it first occurred, and that the preference so established is continued by inheritance to this favored sex.

The whole subject is full of interest, and suggestive of materials for observation and reflection.

IV.—*A Case of Malignant Disease of the Sigmoid Flexure of the Colon.* By T. OSBORNE WALKER, Esq., Jr., M.R.C.S. (Lancet, Aug. 31, 1861, p. 205.)

A case of some interest and rarity is described by Mr. Walker, as commencing with symptoms of enteritis, followed by vomiting, and, on the fourth day from the onset of the inflammatory symptoms, by a copious alvine evacuation, semi-fluid, and of a dark-green color. Signs of enteritis again occurred, but were relieved, until, after a day or two, there was a recurrence of pain, restlessness, tympanites, hiccough, and occasional vomiting; but in two or three days afterwards his medical attendant, being hastily summoned, found him in a state of collapse, exactly twelve hours after the supervention of which he died.

Post-mortem examination revealed a thickened and injected state of the peritoneun generally, with effusion of fæces, and a bruise-like discoloration of the sigmoid flexure of the colon, with several small, darker-colored points of ecchymoses, about the size of, and very much like, leech-bites, in the lowest of which was a small opening of a circular form, and just large enough to allow a hemp-seed to pass through it, through which, on applying pressure, fæcal matter escaped. This opening was situated in the anterior wall of the upper segment of the flexure; and about an inch below this was found a malignant mass, occupying the entire cylinder of the gut for about an inch and a half on the posterior and external wall of the intestine, and binding it down to the iliac fossa so tightly as to occasion great difficulty in its removal. It presented the appearance of cerebriform matter.

The most noteworthy circumstances presented by the foregoing case, and most available for diagnostic purposes, were the small quantity of fluid the bowels was capable of containing without pain and urgent desire to evacuate—not exceeding a pint and a half; the persistence throughout of dullness on percussion over the left iliac region—the site of the disease; the occasional distinct course of the distended colon, appreciable both to inspection and palpation, as far as the diseased portion; the peculiar brownish color of the patient's skin, being quite toadlike; the very small size of the stool once observed; the fluid vomited containing neither bilious nor fæcal matter; neither tumor, stricture, nor ulcer discoverable on examination per rectum, though at the anus was found a hemorrhoidal tumor, of the size of a Spanish nut.

At the onset of the attack, the quantity of urine, which was then voided with difficulty and pain, was small, and of a brandy color, without sediment; but in the course of the case it increased in quantity, and was passed with greater ease, still retaining its deep color. It was ascertained, by inquiry into the history of the case, that the patient had, on some occasions, observed his stools to be slimy and streaked with blood, and had suffered at times much abdominal pain and flatulence, with a bad appetite. The collapse marked the perforation, which was followed by fatal peritonitis.

V.—*On the Symptoms of Organic Disease of the Base of the Brain.* By C. E. Brown-Séquard, M.D., F.R.S. (Lancet, Aug. 31, 1861, p. 199.)

In a lecture forming part of a series on the diagnosis and treatment of the various forms of paralytic, convulsive, and mental affections, considered as effects of morbid alterations of the blood, or of the brain, or other organs, Dr. Brown-Séquard arrives at the following conclusions: From the examination of symptoms caused by organic affections of various parts of the base of the brain, it results that it is chiefly by the symptoms due to the loss of function of the cranial nerves that we may diagnose the precise seat of the alteration. It is most important, therefore, to find out which of these nerves is paralyzed in cases of hemiplegia supposed to depend upon a disease of the brain.

Paralysis of nerves of blood-vessels (the vaso-motor nerves) in organic disease of the base of the brain manifests itself chiefly by a dilatation of the blood-vessels, and an increased temperature, owing to an excess in the quantity of blood. Much remains to be learned on this subject; but certain rules may be laid down. For instance, an elevation of temperature is very frequently observed in the paralyzed limbs in cases of disease at the base of the brain in other parts than the pons Varolii or the medulla oblongata; while, when a hemiplegia depends upon a disease in a lateral half of either of these two organs, (above the decussation of the anterior pyramids,) the increased heat is in the limbs that are not paralyzed.

The following table will show, at the same time, what relates to the paralysis of blood-vessels, and to the paralysis of sensibility and of voluntary movements in cases of diseases of the brain and the spinal cord, limited to a part of a lateral half of these nervous centres:—

*Table of symptoms of loss of function, according to the seat of a lesion in a part of a lateral half of the cerebro-spinal axis.*

1. Lesion in the optic thalamus, the corpus striatum, or one of the cerebral lobes:—

| | On the opposite side. | On the same side. |
|---|---|---|
| Sensibility | Lost. | Normal. |
| Voluntary movements | Ditto. | Ditto. |
| Temperature | Increased. | Ditto. |

2. Lesion in the pons Varolii, or the medulla oblongata, above the decussation of the anterior pyramids:—

| | On the opposite side. | On the same side. |
|---|---|---|
| Sensibility | Diminished or lost. | Increased. |
| Voluntary movements | Ditto. | Normal. |
| Temperature | Diminished. | Increased. |

3. Lesion in the medulla oblongata at the level of the decussation of the anterior pyramids:—

| | On the opposite side. | On the same side. |
|---|---|---|
| Sensibility | Diminished or lost. | Increased. |
| Voluntary movements | Ditto. | Diminished or lost. |
| Temperature | Diminished. | Increased. |

4. Lesion in the spinal cord:—

| | On the opposite side. | On the same side. |
|---|---|---|
| Sensibility | Diminished or lost. | Notably increased. |
| Voluntary movements | Nearly normal. | Diminished or lost. |
| Temperature | Diminished. | Increased. |

This table shows—

1st. That *sensibility* is diminished or lost on the side opposite to that of the lesion, wherever it exists.

2d. That *voluntary movements* are diminished or lost either in the opposite side, or in the side of the lesion, or in both, according to the seat of the lesion.

3d. That *temperature* is increased (owing to the paralysis of blood-vessels) in the side of the lesion when it is in the spinal cord, the medulla oblongata, or the pons Varolii; and in the opposite side when the lesion is in other parts of the cerebro-spinal axis.

It is important to remember that paralyzed blood-vessels may contract under the influence of cold, so that the temperature of a part where blood-vessels are paralyzed may, at times, be very low, especially during the first few months after the beginning of the paralysis.

The symptoms of disease of the base of the brain depending upon irritation, although much more important than the symptoms of loss of function, as they teach much more regarding the nature of the lesion, have been very much less investigated. They may be divided into four distinct series: Symptoms of irritation of motor nervous elements, (nerve-fibres and nerve-cells;) of irritation of sensitive nervous elements; of irritation of vaso-motor or nutritive nervous elements; and of irritation of incito-motor and other special nervous elements.

As a conclusion of the lecture, the author states that it is by the combined examination of the two kinds of symptoms (those depending upon a loss of function, and those due to an irritation) that the seat of an organic affection in a limited part of the base of the brain will be found out. Though there is no great value for treatment in establishing this diagnosis of an organic affection of the brain, there is, at least, an incontestable importance as regards the prognosis in an accurate diagnosis of that kind.

### VI.—*Mortuary Statistics of England.*

1. *Causes of Death in England.* (Lancet, Aug. 17, 1861.)
2. *Deaths in London in* 1660 *and* 1860. (Med. Times and Gaz., Aug. 17, 1861.)

To the Registrar-General's report is appended, as usual, an instructive paper by Dr. W. Farr, on the causes of death in England. The year now reported on (1859) is the first in which diphtheria has obtained a distinct line in the tables; it had previously been confounded with cynanche, and when the two are put together the rapid progress of this great epidemic becomes evident: the deaths in 1855 were 385; in 1856, 603; in 1857, 1583; in 1858, 6606; in 1859, 10,184. Epidemics of diphtheria are clearly described in the seventeenth century by Italian and Spanish writers, and its frequent association with scarlatina justifies the inference that the diphtherine—*ita materies morbi*—is some modification of scarlatinine.

Of the whole deaths of the year, one-fourth were referred to zymotic diseases. Small-pox destroyed 3848 persons, chiefly children, who had not been vaccinated; an instance, as Dr. Farr remarks, of the rigor with which the infringement of sanitary laws is visited, for the children perish, and the parents lose their offspring, by the neglect of a precaution of the simplest kind. A fatal outbreak of erysipelas at the Winchester Infirmary was traced to a cess-pool. Of the parasitic diseases it is remarked that the ova of worms must be derived generally from impure river waters, into which the refuse of towns is poured.

We have but an imperfect conception of the number of deaths from excessive drinking; but 345 were directly ascribed to intemperance, and 545 to delirium tremens—890 in all from the two forms of alcoholism. Passing next to constitutional diseases—another regiment of the enemies that dog our steps—we find gout described as nearly stationary; it is considered that, thanks to the more intelligent system of dining which the wealthier classes, wearied of this racking disease, will probably introduce, we may hope to see gout rapidly decline. The deaths from tuberculous disease have decreased since 1853; those from bronchitis have increased very greatly of late years.

Among local diseases we find affections of the three vital organs—the brain, the heart, and the lungs—causing nearly a third of all the deaths of the year. Fright was the cause of 7 deaths, (not all children,) grief of 8, (7 women,) rage of 5, (4 infants,) anxiety of 1, (a man,) mental shock of 1, (a woman,) melancholy of the deaths of 21 men and 26 women. Above 25,000, chiefly infants, died of convulsions—a striking and distressing symptom—but probably only part of the disease, which is the result of organic lesions and local irritations that are never discovered.

To the decay of old age, without any disease, 27,104 deaths are referred; "the weary wheel of life at length stood still." 14,649 persons were killed—a sad confession, says Dr. Farr, for a nation humane, civilized, and skilled in all the arts, to have to make. Annually 75 persons in 100,000 thus die a violent death. 13,056 of these deaths, in 1859, are ascribed to accident or negligence; among them were 279 by poison. 1248 deaths were declared by coroners' juries to be suicides; 338 murder or manslaughter. 18 persons were killed by lightning, nearly all persons of out-door occupation; the house is safer than the field.

The Registrar-General's report for 1859 contrasts the statistics of disease, so far as we are possessed of them, of two centuries back with those of the present day. It takes 100,000 people of the London of 1660–79, and compares them with the same number of 1859. The same number presented at the two different periods respectively 357 and 42 annual deaths by small-pox; 759 and 227 by fever, ague, scarlatina, quinsy, and croup; 1079 and 611 by consumption and diseases of the breathing organs; 763 and 8 by dysentery; 142 and 2 by scurvy; 298 and 26 by dropsy; 1175 and 136 children's deaths from convulsions and teething, and 86 and 17 women's deaths in child-bearing. All this decrease in the modern figures is over and above the decrease which we gain when we count the deaths of the Great Plague. In one article, however—that of diseases of the brain, apoplexy, paralysis, epilepsy—the older figures have the advantage of the latter, being 57 then to 151 now.

It is deserving of notice, that the only form of disease which has gained ground is that connected with the head, which exceeds now, by nearly 3 to 1, the amount of the same form of disease at the former date, as the figures above show. This is, perhaps, the result of the very civilization and mental growth to which we are indebted for the improvements in every other sanitary quarter.

VII.—*The Small-pox in Prussia in* 1859. (Med. Times and Gaz., Sept. 7, 1861, from the Berlin Med. Zeitung, 1861, No. IX.)

From the report just published on the authority of the Prussian government, we extract the following figures. A great decrease of small-pox occurred in 1859 as compared with 1858; in the latter year there were 30,843 cases, with 2789 deaths; in the former but 16,035 cases, with 1371 deaths. Of the 16,035 cases in 1859, there were 4627 among children, (*i.e.* under 15 years of age,) and 11,408 among adults; and of the 1371 deaths, 569 occurred among children, and 802 among adults—the mortality in the whole number being 8 per cent., among children 12 per cent., and among adults 7 per cent. Of the 16,035 cases, 13,364 had been vaccinated, and 2671 had not been. Of the vaccinated 6 per cent. died; 7 per cent. among the children, and 5 per cent. among the adults. Of the non-vaccinated, 20 per cent. died; 23 per cent. among the children, and 17 per cent. among the adults. Among the cases, 591 had been re-vaccinated, and of these 42 died.

VIII.—*On the Effects of Lightning upon the Human Body.* By DR. W. STRICKER, of Frankfurt. (Virchow's Archiv, Band xx., Hefte 1 and 2, p. 45, quoted in Brit. and For. Medico-Chirurg. Review, July, 1861, p. 262.)

The author of this paper furnishes a detailed description of the mode in which the lightning passes along the body of a person struck, its general results, etc.

He supposes the person injured to have been sheltering under a tree, or to have been exposed openly. In the first case, the lightning in passing from the tree strikes the body on the neck or shoulder, causing a burn and much pain, with extravasation and congestion of the vessels over a broad part of the surface. From this a smaller stripe passes, running down to the nates, gradually getting less, and more superficial, but at the buttocks, where in the man the clothes fit tighter, the conduction is more intense. The lightning is conducted either—(1) by the skin, thence toward the trochanter on one or both sides, the marks becoming weaker, and so on to the knee, where, owing to the tightness of the dress, it causes a deeper burning, runs along the calf, and then, if boots are worn, passes over and destroys them, or passes along the skin to the heel, and wounds it, and after piercing the shoe, makes a hole in the earth; often the lightning passes along the ankle: or (2) it may be conducted along the trousers, which it destroys, or pierces only with a round hole.

When the lightning strikes a person freely exposed on the ground, then the head-covering is destroyed and the vertex smitten. From thence the conduction may be twofold, either (1) from the cranial bones to the brain, producing death by the simple or combined influence of the injury to the brain-mass, or by rupture of the blood-vessels; or (2) along the skin. In the latter case, the skin of the face and neck is almost always completely spared, and the lightning effects a considerable burn over the sternum; in some cases it enters the mouth, affects the teeth and tongue, causes bronchitis, loss of voice, etc. Proceeding downward, the track passes toward the inguinal region, only the shirt being sometimes torn, and then an interruption of conduction often occurs, occasioning deep burning of the groins, genitals, etc.; and occasionally we have mortal laceration of the intestines, or in milder cases injection of the liver, spleen, stomach, etc. The conduction, then, is through the skin or clothing, or both, to the back of the foot, where a wound is produced. The burning of the hair is very remarkable, and this may happen without any injury to the skin.

The burning produced by the lightning varies from that of the cautery to a mere drying of the tegumental surface.

The general results of injury by lightning are:—

I. In those killed, the rapid supervention of putrefaction and the dilatation of the pupils. The hemorrhage from the nose and mouth is not sufficiently understood.

II. In those only outwardly injured, (partly, perhaps, from fright.)

1. Excessive stupefaction.

2. Great alternate depression and exaltation, long-drawn inspiration, small, slow pulse, cooling of the skin, muscular debility.

3. Suppression of urine, and constipation; vomiting, loss of appetite, sometimes purging.

4. Great painfulness of the part affected, which increases for two days, and then declines.

5. On the uterus of pregnant women no peculiar effect is produced; like any other fright, it tends to arrest existing menstruation.

6. Cataract and nyctalopia may possibly be a result.

IX.—*Hypertrophy of the Heart, with Ossification of the Aortic Valves.* Post-mortem examination of the body of the late Lord Chancellor Campbell. Communicated by WILLIAM ACTON, ESQ. (Quoted in the Dublin Medical Press, Sept. 4th, 1861, from the London Lancet.)

On the 24th of June, Mr. Acton was requested to make the necroscopic examination of the late Lord Campbell. It appears that the subject of this notice, after retiring to bed in his usual health at half-past twelve o'clock at night, was found dead at eight o'clock the next morning; and the *post-mortem* was made thirty-four hours after the dead body was discovered. The heart and aorta were found seriously affected, and we may therefore exclude from special consideration other appearances which were not of such material importance.

*Heart.*—Large, weighing twenty-five ounces, (the usual weight being eight or nine ounces,) firm, with a deposit of fat on the surface of the right ventricle. Thickness of the walls of the left ventricle about three-quarters of an inch. The structure of the organ was unusually firm, considering the age of the deceased peer, (eighty-one years.) Some deposit of fat beneath the endocardium. The cavities contained small quantities of dark, fluid blood; no clots whatever.

*Aorta.*—Lining membrane stained. Aortic valves, although not much thickened at their edges, were incrusted on their aortic aspect with calcified deposits of intense hardness. The deposit could not be cut, but might be broken. A similar incrustation was noticed on the outer surface of the middle aortic valve. The arch of the aorta was very much stained, and presented numerous but small patches of atheromatous deposits. Similar appearances were noticed at the roots of the vessels arising from the aorta. No evident atheroma of the coronary arteries, which were carefully traced.

The only structure which could be said to have become degenerated, after sixty-five years of hard and almost uninterrupted labor in law, letters, and politics, was that of the aortic valves. These, as the *post-mortem* examination demonstrated, were thoroughly changed in structure, and that this was the immediate cause of his death will be presently shown.

Although the heart was nearly three times the normal weight, its structure for a man of eighty was unusually sound, and, what was equally remarkable, the coronary arteries which supplied the heart itself with blood, and which one might have fully expected to find diseased, had evidently been quite recently fulfilling their due functions. It was owing, then, probably, to the possession of a heart so organized, a remarkably large, firm, healthy brain, capacious lungs, and an uninjured liver, that the functions of the late lord's life were so long and uninterruptedly carried on, in spite of a pathological condition of the aortic valves, which would in ordinary cases have long since been fatal, and which eventually determined this one.

It appears, from the statements of the family, that the late lord had enjoyed through his long life excellent health. He rarely, if ever, had occasion to consult medical men; and neither he nor his family had any idea of his ever having suffered from any disease of the heart, rheumatism, or fainting fits. He had been in the daily habit of walking from the House of Lords to his home. He could mount stairs without palpitation. He was strictly abstemious, and on the evening preceding his death had not taken any indigestible food. The *post-mortem* examination enables us with certainty to infer the way in which death took place. After retiring to his bedroom, his lordship undressed and went to bed. After some time, (it is impossible to conjecture how long,) probably finding himself faint, he rose, threw off his body linen to obtain relief, and was about to sit down in the easy chair, when a fainting fit seized him. He became unconscious, having previously coughed up the matter found on his shirt, and he remained in that position until found by his valet at eight A.M.

The *physical cause* of death was probably as follows: Having labored for some years under ossification of its aortic valves, the heart (as is not unusually the case, even without any assignable cause) becomes sooner or later fatally embarrassed in its action. This embarrassment of circulation, it may be inferred, came on without warning, after the deceased had lain down on the morning of the 23d of June, and, producing a feeling of faintness, prompted him to rise from his bed. This effort was of no avail; for the lungs, also engorged, were unable to relieve themselves, and through their failure, the brain, becoming speedily charged with unoxygenated blood, ceased at once to perform its functions. We must presume that the subarachnoid effusion, as well as that into the cavity of the pleura, took place nearly one or two hours before death.

X.—*The Diseases of Hong Kong and the Canton River Station.* By Dr. Smart. (Lancet, Aug. 3, 1861, p. 114.)

From the analysis, contained in the *Lancet*, of a paper on the relation of Chinese climate to the diseases prevalent, derived from the statistics of the Royal Naval Hospital at Hong Kong, during thirteen years, we make the following condensed extracts:—

*Climatic diseases.*—621 cases are quoted under the head of fevers, 83·25 per cent. of which were cured; 10·46 per cent. invalided; and 5·95 per cent. died. Of 1471 cases of intestinal disease, 58·12 per cent. were cured; 24·67 per cent. invalided; and 16·65 per cent. died. Of liver diseases, 57 per cent. recovered; 24·3 per cent. were invalided; and 18·69 per cent. died. Spring is the healthy season; June, July, and August, and October to January, being unhealthy months, those being the periods when the plains are undergoing artificial drainage for the rice harvest, or when the whole surface is being dried by spontaneous evaporation. Deaths from fevers are fewer in the worst season, the type of fever and other causes producing such a paradoxical result.

*Small-pox.*—Inoculation is practiced in the spring, so that the disease is epidemic at that time. In the propagation of cow-pox from imported virus, Dr. Smart found it of uncertain operation.

*Diseases of the abdominal viscera* were most intense in the months of falling temperature, with a drying soil, from October to January, inclusive; and again in the hot, moist months, from June to August. In the thirteen years of Dr. Smart's investigations, there was but one cholera season. When peritonitis prevailed, pleuritis was also frequent.

A peculiar form of bowel-complaint occurs in China, known as "diarrhœa acholica," on account of the yeasty or pipe-clay stools; as diarrhœa with marasmus, or as diarrhœa with debility, and occasionally as "diarrhœal dysentery;" and, in India, as "diarrhœa alba," and "hill dysentery." It differs from tropical dysentery in not being a disease of deposit, but simply one of nutrition, from non-absorption of aliments, depending upon functional derangement of the chief absorbents, the spleen, and the liver.

*Liver diseases.*—The ratios of mortality and invaliding in liver diseases are found to be almost equal to those of dysentery, the mortality being 20·4 and 20·8 per cent., and the invaliding 26·5 and 25·47 per cent., in the two diseases respectively.

*Fevers.*—When the zymotic causes predominate, as in the summer and autumnal fevers of 1858 and 1859, among the troops in garrison at Canton, the typhomania and rose-colored eruption which characterize "typhoid," are truly pathognomonic of the lesions met with in the autopsies. The history of such cases removes all doubt of the existence of the zymotic causes of typhoid in the southern cities of China, as in the crowded parts of the cities of Europe.

*Entozoa* are of frequent occurrence; *diseases of the nervous system* were more frequent, and their mortality highest in the Christmas quarter; *hydrophobia*, as a consequence of "rabies canina," is known throughout China; *leprosy* exists in the same form as that which is seen in the Levant, while those affected are considered outcasts, and obtain their livelihood by begging.

*Affections of the respiratory organs* are not frequent enough to be of primary climatic importance, but the fatality, etc. indicates more than the average severity of type. In regard to the results of the pulmonary complications of the climatic fevers, Dr. Smart thinks pneumonia, as a complication of fever, was less fatal, and pleurisy more fatal, than when of idiopathic origin.

*Syphilis.*—The syphilitic cachexy is most difficult of eradication from the system in China, and has a marked influence on the effects of treatment in the climatic diseases, more especially in dysentery. Nearly 16 per cent. of the nosological table were syphilitic cases. Periosteal disease, as a consequence of syphilis, forms a large group in the class of cases arranged under the head of rheumatism, though some of the invalidings from the latter also include implications of the heart in arthritic cases.

*Diseases of the integuments and glands* contributed 17 per cent. of the total cases of disease. Phlegmon is a most distressing complaint to almost all new-comers in the spring and summer. An attack of boils is borne patiently, under the conviction of the sufferer that it wards off climatic diseases. Boils are often preceded by "lichen tropicus," another imputed salutary effort of milder form. Abscesses are often of a grave character, a mortality of 7 per cent. being an indication of their severity; and Dr. Smart is disposed to consider acute suppuration of the areolar tissue as the ordinary cause, often setting in on the subsidence of fever.

XI.—*The Hot Bath: its Physiology, Use, and Abuse; especially in connection with the Treatment of Drowning.* By CHARLES HUNTER, Esq., M.R.C.S., etc. (Lancet, Aug. 10, 1861.)

In a previous number of this Journal we referred to the use of the hot bath in cases of asphyxia from drowning. After remarking that if in drowning, the failure of cardiac action was the main evil and the cause of the other symptoms, then the employment of the hot bath might be advantageous, but that if the failure of the heart's action was a secondary, or brought-about phenomenon, then the warm or hot bath—a question only of degree—would be certainly prejudicial, Mr. Hunter proceeds to inquire whether, in drowning, vitality ceases from the lungs first, or from the heart. It can be shown and verified, he says, that—

1st. In drowning, the lungs are the organs first and mainly affected.

2d. That the heart is influenced only in a secondary and consequent manner. And that, therefore, with regard to the mode of death—

3d. The lungs cease to act before the heart does; and

4th. Death is from the lungs by apnœa, or its secondary effects on the system. From which established fact it stands to reason—

5th. That the first and chief part of the treatment should be directed to the lungs and the improvement of the pulmonic circulation; and

6th. That the heart and systemic circulation should only receive secondary attention.

After quoting the views of authorities, Goodwyn, Fothergill, Taylor and others, the writer draws this inference: That the longer cardiac pulsations continue, after the lungs have ceased to act and purify the blood, the more effectually is the system being narcotized by the non-eliminated carbonic acid in the blood. And with this important truth, as he calls it, firmly fixed in his mind, he condemns the use of the hot bath for the purposes of stimulation in cases of drowning as pernicious and reprehensible.

Submersion or drowning, as to its effects, may be thus given:—

*Main effects of submersion.*

Occasioning primary apnœa:

1. Deprivation and induced expiration of air; cessation of lung action.
2. Embarrassed cardiac action; embarrassed blood circulation.

*Secondary effects.*

Occasioning secondary apnœa:

3. Absence of blood purification.
4. Induced absence of nervo-muscular power; of consciousness, reflex action, etc.
5. Gradual loss of animal heat.
6. Entire cessation of cardiac action.

XII.—*On the Pathology of Asthma.* By G. H. Kidd, M.D., Assistant Physician to the Coombe Lying-in Hospital, Dublin. (Dublin Quarterly Journal of Medical Science, May, 1861, p. 292; quoted in British and Foreign Medico-Chirurgical Review, July, 1861, p. 263.)

The physiological and pathological conclusions arrived at by Dr. Kidd are as follows:—

1. During the paroxysm of asthma, the chest is distended to the greatest possible extent. 2. The muscles of inspiration are in spasmodic action, (tonic spasms.) 3. The bronchial muscles are muscles of inspiration, and associated in the spasmodic action with the other muscles of inspiration. 4. Breathing is carried on by the voluntary effort to aid the muscles of expiration, and as soon as this is relaxed, the muscles of inspiration, like so many stretched bands of india-rubber, distend the chest again.

That the spasm of the bronchial muscles in asthma arises from some morbid action in the medulla oblongata, is to be inferred from the following facts:—

1st. The fact that the spasm affects an entire group of muscles. Now, Schroeder Van der Kolk has shown that muscles associated in action are supplied by nerves arising from special groups of mutually associated and connected ganglion corpuscles. Disorder of this group would then manifest itself in the entire class of muscles.

2d. Van der Kolk has also shown that the skin covering parts moved by muscles is supplied with sensitive nerves, arising from the same segments of the spinal centre as the motor nerves of those muscles arise from. Dr. Salter has remarked, as an almost universal symptom of asthma, that there is itching of the skin under the chin, over the sternum, and between the scapulæ. This, it is evident, is a subjective sensation, and indicates an irritation existing at the roots of the nerves.

3d. Paroxysms of asthma are observed to occur in cases of acute hydrocephalus, as in a case mentioned by Dr. Salter, and in one mentioned by Dr. Graves, where there were also general convulsions. In persons liable to epilepsy recurring at regular intervals, fits of asthma occasionally take the place of, or serve as a substitute for, the epileptic fits.

4th. The state of the patient producing the fit of asthma indicates an affection of the nervous centres. In one there is mental exhilaration, in another mental depression. A patient of Sir J. Forbes is awakened by convulsions in one foot and leg, and as soon as the asthmatic fit is developed, the convulsions of the extremity cease.

5th. The exciting cause indicates the same. In one, cold water applied to the instep will cause an attack; in another, sudden emotion, going to bed with a loaded rectum, etc. etc. Emotion will also check the paroxysm when fully developed. Hence it may be inferred that asthma depends on a morbid state of the medulla oblongata and spinal centres, which manifests itself by throwing the entire group of inspiratory muscles into spasmodic action.

